# Power to participants: a call for person‐centred HIV prevention services and research

**DOI:** 10.1002/jia2.25167

**Published:** 2018-10-18

**Authors:** Marija Pantelic, Christine Stegling, Sally Shackleton, Enrique Restoy

**Affiliations:** ^1^ International HIV/AIDS Alliance Brighton & Hove United Kingdom; ^2^ Department of Social Policy and Intervention University of Oxford Oxford United Kingdom; ^3^ International HIV/AIDS Alliance Cape Town South Africa; ^4^ School of Global Studies University of Sussex Sussex United Kingdom

**Keywords:** person‐centred, HIV, prevention, participatory research, community‐based organisations

## Abstract

**Introduction:**

While biomedical HIV prevention offers promise for preventing new HIV infections, access to and uptake of these technologies remain unacceptably low in some settings. New models for delivery of HIV prevention are clearly needed. This commentary highlights the potential of person‐centred programming and research for increasing the cultural relevance, applicability and use of efficacious HIV prevention strategies. It calls for a shift in perspective within HIV prevention programmes and research, whereby people are recognized for their agency rather than assumed to be passive beneficiaries or research participants.

**Discussion:**

Person‐centred HIV prevention reorientates power dynamics so that individuals (rather than interventions) are at the centre of the response. Respecting personal choice and agency – and understanding how these are shaped by the context in which people exercise these choices – are critical dimensions of the person‐centred approach. Community‐based participatory research should be employed to inform and evaluate person‐centred HIV prevention. We argue that community‐based participatory research is an orientation rather than a method, meaning that it can be integrated within a range of research methods including randomized controlled trials. But embracing community‐based participatory approaches in HIV prevention research requires a systemic shift in how this type of research is reported in high impact journals and in how research impact is conceived. Community‐based organizations have a critical role to play in both person‐centred HIV prevention and research.

**Conclusions:**

HIV prevention is situated at the intersection of unprecedented opportunity and crisis. Person‐centred approaches to HIV prevention and research shift power dynamics, and have the potential to ensure a more sustainable response with each individual actively participating in their own care and meaningfully contributing to the production of knowledge on HIV prevention. This approach taps into the resourcefulness, resilience and knowledge of the person and their communities, to strengthen research and programmes, making them more relevant, appropriate and effective.

## 1 Introduction

Biomedical HIV prevention research has made a major breakthrough, making the end of HIV possible, at least in theory. It has been established that antiretroviral treatment (ART) is an efficacious HIV prevention tool 1 for people living with HIV who have undetectable viral loads. Moreover, the use of pre‐exposure prophylaxis (PrEP) by people not living with HIV pre‐emptively inhibits HIV acquisition 2. The combination of HIV prevention interventions and strategies has led to an overall worldwide decline in new HIV infections: In 2016 there were approximately 1.6 million new HIV infections among people over 15 years, a reduction of 10.6% compared to 2010 3.

But this decline is far from the prevention target that most governments pledged to achieve when they signed the 2011 Political Declaration on HIV and AIDS. The target was a 50% reduction in new infections acquired through sexual transmission or injecting drug use between 2010 and 2015 4. Social and structural factors continue to compromise access to and use of evidence‐based biomedical HIV prevention strategies among populations most affected by HIV 5, 6, 7. Indeed, approximately 45% of all new seroconversions globally are among sex workers, gay, bisexual and other men who have sex with men and people who inject drugs 3. These rates have either remained steady or increased over the years.

New models of delivery of HIV prevention are clearly needed to ensure that nobody is left behind. In this commentary we highlight the potential of person‐centred programming and research for increasing the cultural relevance, applicability, efficacy and uptake of HIV prevention strategies 8, 9. We suggest key areas for consideration to help shape HIV prevention services and research. We do not provide a specific set of guidelines because person‐centred HIV prevention services and research are context‐specific and highly dependent on individuals’ preferences, concerns and needs 10. Rather, we call for a shift in perspective within HIV prevention programmes and research, whereby people are recognized for their agency rather than their vulnerabilities.

## 2 Discussion

### 2.1 Applying a person‐centred lens to HIV prevention

There is an increasing recognition that HIV prevention must be reorientated so that it places people (rather than interventions or disease) at the centre of our response 10, 11. Person‐centred HIV prevention is a principled approach 12, which builds on the Greater Involvement of People living with HIV (GIPA) principles and the Positive Health, Dignity and Prevention Framework 13 to offer an inclusive model for HIV prevention services, which can otherwise sometimes overlook their users’ complex needs. However, person‐centred HIV prevention also corresponds to evidence on HIV epidemiology, health service research 9 and a public health perspective, which recognizes that people living with HIV and those at risk of acquiring the virus are deeply affected by socio‐economic, legal and cultural environments, which in turn affects their enrolment and continued engagement in HIV prevention, treatment and care 6, 7, 14. In addition to acknowledging that socio‐environmental factors shape people's decisions and health outcomes, person‐centred services aim recognize and respond to people's needs and competencies 15.

At the core of person‐centred HIV prevention is the acknowledgement that people are best placed to decide which prevention methods are right for them 4. Person‐centred HIV prevention also recognizes that a person's health needs change over the course of their life 10. A person's needs are also shaped by a range of factors that are personal (age, gender, gender identity, profession, etc.), contextual (location, community, physical security, economic status, etc.) and structural (stigma, racism, violence, criminalization, political and legal participation). By investing in long‐term relationships with people and their communities we can sustain their involvement and make space for demand‐driven services and community action to hold policy makers to account to end AIDS. Respecting personal choice and agency – and understanding how these are shaped by the context in which people exercise these choices – are critical dimensions of the person‐centred approach. The evidence base on person‐centred HIV prevention is in very nascent stages, particularly in low‐ and middle‐income countries which bear the brunt of the HIV epidemic. However, the broader literature on healthcare suggests that person‐centred services hold promise for people's health outcomes. For example, a recent systematic review examining the efficacy of person‐centred care as an intervention in controlled trials found that 8 out of 11 included studies showed person‐centred care to be successful 9.

While person‐centredness 16 is not a new concept, adapting the delivery of HIV programming to individual needs is a departure from intervention and risk‐focused approaches. It should be noted that differentiated services have begun to shift focus to more responsive and customized offerings. However, they categorize (and sometimes assume) people's needs based on treatment status or age 17. Differentiated services are an important step in the right direction to addressing people's diverse needs but they are still intervention focused, and categorize people based on their level of risk. While a differentiated service is oriented around the needs of epidemiologically relevant subgroups of people 17, a person‐centred service aims to respond to an individual person's needs, which may vary over the course of their life 10.

Evidence on person‐centred HIV prevention programming is scarce but emerging studies suggest it may help reach the most marginalized populations who may have intersecting vulnerabilities and are not being reached through public health systems. For example, Women Initiating New Goals of Safety (WINGS) is an individualized screening, brief intervention and referral to treatment model for addressing intimate partner violence and HIV risks among women who use drugs or engage in heavy drinking 18. Following a harm reduction approach and Social Cognitive Theory, WINGS aims to employ a ‘non‐judgmental stance to meet women where they are with respect to their intimate relationships and to enable them to set and enact their own goals to improve relationship safety based on whether they wish to stay with or leave their partners’ 18. The model includes individual tailoring to women's needs and boundaries, identifying individual motivation for behaviour change and the manual requires facilitators to build on individual women's strengths. Based on the information provided, facilitators identify existing ways in which women who use drugs have developed personalized coping strategies, solved problems and exhibited courage and determination 18. Recent randomized controlled trials suggest that the programme is effective in reducing various forms of gender‐based violence experienced by women who use drugs in the United States 19 and Kyrgyzstan 20, which is likely to have follow‐on effects on HIV prevention 21. In India, a preliminary pilot suggested that the intervention is feasible when delivered by other women who use drugs, and a pre–post evaluation indicated reductions in intimate partner and other violence victimization 22. Together with HIV/AIDS Alliance India, we are currently planning a randomized trial to examine whether this person‐centred intervention brings added benefits to regular harm reduction for women who use opioids in India.

There is an urgent need for more evidence on which person‐centred approaches work for whom and in what contexts, and for evidence‐informed implementation guidance. The following sections of this paper highlight the need for person‐centred HIV prevention research to meaningfully engage with communities and call for a shift in how community participation in HIV prevention research is reported.

### 2.2 Implications for person‐centred HIV prevention research

#### 2.2.1 Community‐based participatory research and re‐orientating the locus of power in research

Person‐centred research is determined based on the *focus of enquiry*: it is defined as research examining person‐centredness 23. We posit that community participatory action research is an adequate *orientation* for developing or evaluating HIV prevention interventions that aim to be person‐centred.

Community‐based participatory research involves planning, executing and disseminating research “*with* the people whose life‐world and meaningful actions are under study” 24. The main difference between participatory and non‐participatory research is the locus of power and ownership of the research process 24. Participatory research places its participants at the centre of the knowledge production process. This perspective recognizes that the validity and applicability of research findings are highly dependent on meaningful involvement of community expertise. A growing evidence base on participatory research sets a strong foundation for guiding people on various practical aspects of meaningful engagement of communities in HIV prevention research. Drawing on practical experience, researchers have reported on the benefits and challenges of co‐designing interventions, building capacity so that community partners understand the utility of evidence for advocacy and setting funding priorities, and using participatory research to comprehend the cultural acceptability and applicability of HIV prevention tools 25, 26, 27, 28. UNAIDS and AVAC published Good Participatory Practice guidelines for biomedical HIV prevention trials, which recommend community participation to strengthen the ethical and scientific quality of biomedical HIV prevention trials 29. However, to our knowledge, there is no similar consolidated set of guidelines for community participation in non‐biomedical HIV prevention research.

Building further from the aforementioned participatory practices, if a study is concerned with also being *person‐centred*, then the focus of enquiry must expand from a disease (or vulnerability to the disease) to the whole person and their lived experience 15, 30. As part of this, person‐centred research explicitly examines people's integration within their environment, their relationships with other actors in their lives, their aspirations and their rights 9. In practical terms, this means that while all person‐centred research is participatory, not all participatory research is person‐centred. For example, it is possible for a study concerned with biomedical HIV prevention to follow good participatory practice guidelines but focus only on clinical outcomes determined based on a person's HIV risk 29. In contrast, a person‐centred study would also examine the wider aspects of people's everyday lives that might have the potential to strengthen HIV prevention 30, 31. HIV prevention studies mainly measure HIV prevention outcomes such as condom use, reduction in viral loads and PrEP use. However, from a person‐centred perspective, outcomes measured should reflect what matters to service users, even if this entails a departure from what is normally considered as relevant to public health, for example, sexual pleasure outcomes 32. Critical to person‐centred research is anti‐reductionism and a commitment to understanding people's strengths, potential and resilience 15.

Person‐centred research is grounded in the belief that the evidence on HIV prevention must adequately respond to the broad needs and aspirations of people who take part in the research and who we hope to uptake the HIV prevention technologies and interventions. For example, a mixed‐methods longitudinal study of adolescents living with and affected by HIV in South Africa, has used a participatory approach to examine what might improve young people's uptake of health services. Through the “dream clinic” exercise 33, a qualitative method which was co‐developed with adolescents, young people designed and drew their ideal health facilities. The resulting “dream clinic” illustrations were analysed together with young people. Findings indicated a wide range of aspirations that young people have for their health services, including clean water supplies and food through soup kitchens, tuck shops and/or gardens. Young people also expressed their desire for easily accessible healthcare, with well paved roads, proximity to their homes and schools and linkages to social services. Their dream clinics included healthcare providers who treated them respectfully. This person‐ centred and participatory research study produced practicable recommendations for innovations in development and healthcare, and informed the objectives of South Africa's 2017 National and Adolescent and Youth Health Policy.

#### 2.2.2 Researchers should be accountable to communities they aim to serve

Participatory research has often been categorized as a qualitative research method – portrayed in contrast to positivist quantitative science 34. We position person‐centred research as an orientation rather than a method, meaning that it is compatible with and can be employed in quantitative HIV prevention research 35. Even randomized controlled trials, which are considered the golden standard of evidence, can be conceptualized, designed and implemented through community‐based participatory partnerships 36. For example, within a community based participatory partnership, Rhodes and colleagues 37, tested an HIV prevention intervention with and for immigrant Latino men who have sex with men in the United States. Essential to this process was capacity building among community partners to understand the utility of high‐quality evidence for policy change and for guiding funding priorities 37. Unfortunately, there are few HIV prevention studies that report employing both a quasi‐experimental or experimental design and community‐based participatory approaches 34. Reasons for this remain unknown because, as noted above, applying community‐based participatory approaches to robust quantitative studies is possible. Evidence from broader HIV‐related research further supports the notion that participatory research methodologies can be applied to quantitative studies. For example, Mavhu and colleagues have used mixed methods participatory research to highlight the dominant issues in the lives of young people living with HIV in Zimbabwe, using it to enhance existing adherence and sexual and reproductive health programming with psychosocial support 38. Person‐centred HIV prevention is possible only if the production of knowledge is co‐owned between researchers and the community. In line with this, we reiterate that community‐based participatory research can and should be applied across the spectrum of research methods.

Embracing community‐based participatory approaches in HIV prevention research requires a systemic shift in how this type of research is reported in high‐impact journals. High impact peer‐reviewed publications featuring emerging evidence on HIV prevention, including this journal, require that authors adhere to gold standard reporting guidelines for effectiveness and epidemiology studies. But the relevant reporting guidelines for randomized controlled trials 39, 40 and observational studies 41 do not include requirements to report on community involvement in the research. Quantitative HIV prevention studies may employ community‐based participatory approaches more frequently than is reported. However, without proper documentation readers are not able to understand or evaluate to what extent this has occurred, and are not capacitated to replicate approaches to community‐based participatory research 42. Leading multidisciplinary HIV and AIDS journals such as this one are uniquely positioned to catalyse a culture change in how quantitative HIV prevention research is conceived and reported.

Further, for those of us providing HIV prevention services and strategies, the outcome of community‐based participatory research cannot be stand‐alone research outputs. Rather, the research process should be fully embedded in and intertwined with all other elements of HIV prevention. For us, HIV prevention research is a tool for optimizing service delivery. In order to inform person‐centred HIV prevention, the research must also be participatory, whereby people are not merely participants but rather essential technical advisors, partners in the research design and implementation, co‐owners of data and key stakeholders for dissemination 12.

Networks of key populations and people living with HIV, community groups, women's rights groups and community activists can play instrumental roles in posing difficult ethical questions, identifying relevant community partners and helping ensure that the research is conducted in a way that maintains accountability to communities. Community‐based participatory research in the context of HIV is challenging. Debates around these challenges are important and, in our view, reinforce the importance of engaging with community‐based organizations in HIV prevention research. For example, researchers have expressed tensions between the basic tenets of ethics to protect participants versus the basic principles of community‐based participatory research which recognizes people's autonomy and authority over their own lives 43. Questions have also been raised around who represents the community 34? Community‐based organizations working on the frontlines of HIV prevention and human rights have an essential role to play in defining ethical guidelines for this type of research. Without the possibility to engage all members of an affected population, community organizations can provide critical linkages, offer guidance for meaningful engagement, and be a vital source of real‐time data about the issues the population is facing.

## 3 Conclusions

HIV prevention is situated at an intersection of unprecedented opportunity and crisis, with prevention targets not being met for marginalized populations 3, 4. While biomedical HIV prevention offers promise for reducing the spread of HIV, access to and uptake of these technologies remain unacceptably low in many settings. Key populations disproportionately affected by HIV continue to experience severe structural barriers to HIV prevention, including stigma and criminalization 6, 44. Few issues in the HIV response are more urgent than to apply a more person‐centred approach to prevention for these communities. Ultimately key populations have a wealth of experience in manoeuvring their lives and they know exactly what is appropriate and effective in their circumstance. Person‐centred HIV prevention services should listen and respond to these perspectives.

In order to achieve this, a reorientation of power dynamics in research is essential. We posit that community‐based participatory approaches to research are highly relevant to shaping person‐centred HIV prevention. Here, community‐based participatory research is employed as an orientation to scientific enquiry, which can be applied to both qualitative and quantitative research methods. Community‐based organizations have a critical role to play in strengthening community–academic partnerships and ensuring that research is done ethically in a way that is accountable to communities.

Person‐centred approaches to HIV prevention services and research shift power dynamics, and have the potential to ensure a more sustainable response with each individual actively participating in their own care. This approach taps into the resourcefulness, resilience and knowledge of the person and their communities, to strengthen research and programmes, making them more relevant, appropriate and effective.

**Table:** 

Key recommendations for person‐centred HIV prevention and research
**Recommendations for programme implementers** Recognize that there is no one‐size‐fits‐all solution and be willing to implement flexibly Treat people as experts, not patients Recognize that people are resourceful, learn about the strategies they use to improve HIV prevention and capitalize on this
**Recommendations for researchers** Use participatory approaches to designing, implementing and reporting on research so that communities’ preferences are taken into account. This applies to both qualitative and quantitative studies. Investigate research questions that highlight people's strengths and aspirations rather than just risks and vulnerabilities When writing a paper, report on community engagement; when reviewing a paper, ask authors to report on it; when editing a journal or special issue, make it a requirement for empirical papers to report on community engagement (or lack thereof).

## Competing interests

The authors declare that they have no competing interests.

## Authors’ contributions

CR, ER, MP and SS conceptualized the commentary. MP, CR and SS provided content on person‐centred HIV prevention programming. MP and ER provided content on implications for research. MP drafted the manuscript, and all authors contributed to revisions.
